# Automated segmentation of human cervical-supraclavicular adipose tissue in magnetic resonance images

**DOI:** 10.1038/s41598-017-01586-7

**Published:** 2017-06-08

**Authors:** Elin Lundström, Robin Strand, Anders Forslund, Peter Bergsten, Daniel Weghuber, Håkan Ahlström, Joel Kullberg

**Affiliations:** 1 Department of Radiology, Uppsala University, Uppsala, Sweden; 2 Department of Information Technology, Uppsala University, Uppsala, Sweden; 3 Department of Women’s and Children’s Health, Uppsala University, Uppsala, Sweden; 4 Children Obesity Clinic, Uppsala University Hospital, Uppsala, Sweden; 5 Department of Medical Cell Biology, Uppsala University, Uppsala, Sweden; 6 Department of Paediatrics, Paracelsus Medical University, Salzburg, Austria; 7 Obesity Research Unit, Paracelsus Medical University, Salzburg, Austria; 8 Antaros Medical, BioVenture Hub, Mölndal, Sweden

## Abstract

Human brown adipose tissue (BAT), with a major site in the cervical-supraclavicular depot, is a promising anti-obesity target. This work presents an automated method for segmenting cervical-supraclavicular adipose tissue for enabling time-efficient and objective measurements in large cohort research studies of BAT. Fat fraction (FF) and R_2_^*^ maps were reconstructed from water-fat magnetic resonance imaging (MRI) of 25 subjects. A multi-atlas approach, based on atlases from nine subjects, was chosen as automated segmentation strategy. A semi-automated reference method was used to validate the automated method in the remaining subjects. Automated segmentations were obtained from a pipeline of preprocessing, affine registration, elastic registration and postprocessing. The automated method was validated with respect to segmentation overlap (Dice similarity coefficient, Dice) and estimations of FF, R_2_^*^ and segmented volume. Bias in measurement results was also evaluated. Segmentation overlaps of Dice = 0.93 ± 0.03 (mean ± standard deviation) and correlation coefficients of r > 0.99 (P < 0.0001) in FF, R_2_^*^ and volume estimates, between the methods, were observed. Dice and BMI were positively correlated (r = 0.54, P = 0.03) but no other significant bias was obtained (P ≥ 0.07). The automated method compared well with the reference method and can therefore be suitable for time-efficient and objective measurements in large cohort research studies of BAT.

## Introduction

Brown adipose tissue (BAT) is considered as a promising target for treatment of obesity and diabetes^1^ due to its high capacity of converting chemical energy into heat^2^. Positron emission tomography combined with computed tomography (PET/CT) using [^18^F]fluorodeoxyglucose (FDG) is currently the most established modality for *in vivo* imaging of human BAT^3^, of which the main depot is located in the cervical-supraclavicular region^4^. As a non-ionizing imaging alternative and complement to PET/CT, magnetic resonance imaging (MRI) has been introduced for studies of human and animal BAT during the metabolically active and inactive states^5–11^. One promising MRI technique, multi-echo water-fat MRI, can provide quantitative and simultaneous estimations of fat fraction (FF) and R_2_^*^^12^. The FF and R_2_^*^ metrics might reflect differences between BAT and white adipose tissue (WAT), with respect to water, fat, iron and blood oxygen content, and have been used for characterizing the two types of adipose tissue (AT)^13^.

An important step in BAT image analysis is segmentation of the BAT depots. In human FDG-PET/CT studies, isolation of metabolically active BAT is guided by the use of combined constraints on FDG standardized uptake value (SUV) and CT Hounsfield units (HU)^3^. In studies involving separate examinations with FDG-PET/CT and MRI^14^ or simultaneous examinations with integrated FDG-PET/MRI^15^, segmentation of the MR images can be accomplished by using the co-registered PET/CT data or PET data, respectively. In standalone MRI studies, there is so far no reference available for identifying active BAT or BAT amount. Instead, these studies typically rely on manual segmentation, based on anatomical criteria, for targeting AT with high *a priori* probability of containing BAT. The cervical-supraclavicular adipose tissue depot is an example of such *suspected* BAT and is hereafter denoted sBAT (for illustration of the depot location, see previous publication by Enerbäck^16^). BAT depots in human adults are not well-defined as compared to the traditional subcutaneous and visceral WAT depots^17^ or the characteristic interscapular BAT (iBAT) depot in rodents and human infants^18^. The extension of the sBAT depot is relatively subjective and may cover a smaller or larger portion of the non-subcutaneous adipose tissue present in the cervical-thoracic region. Besides the challenging task of anatomically defining the sBAT depot in a standardized manner, manual delineation suffers from general drawbacks such as operator expertise dependency, limited precision, time-inefficiency and high cost^19^. A fully automated segmentation method would facilitate MR image analysis and lead to standardized, reproducible and objective measurements suitable for large cohort studies. Multi-atlas segmentation (MAS) is a promising automated method for BAT segmentation due to its state of the art performance for diverse applications and suitability for data exhibiting large anatomical variations^19^. Moreover, MAS methods have successfully been implemented on water-fat MRI data for the purpose of studying volumes of muscle^20^ and adipose tissue^21^. The basic concept of MAS is to use a group of previously segmented datasets (atlases) to segment a novel dataset (target) via image registration.

The purpose of the present work was to develop and validate an automated multi-atlas method for segmentation of human cervical-supraclavicular adipose tissue (sBAT), intended for studies of brown adipose tissue (BAT) in large cohort studies. The validation was performed by using a semi-automated segmentation method as reference.

## Materials and Methods

**Figure 1:**
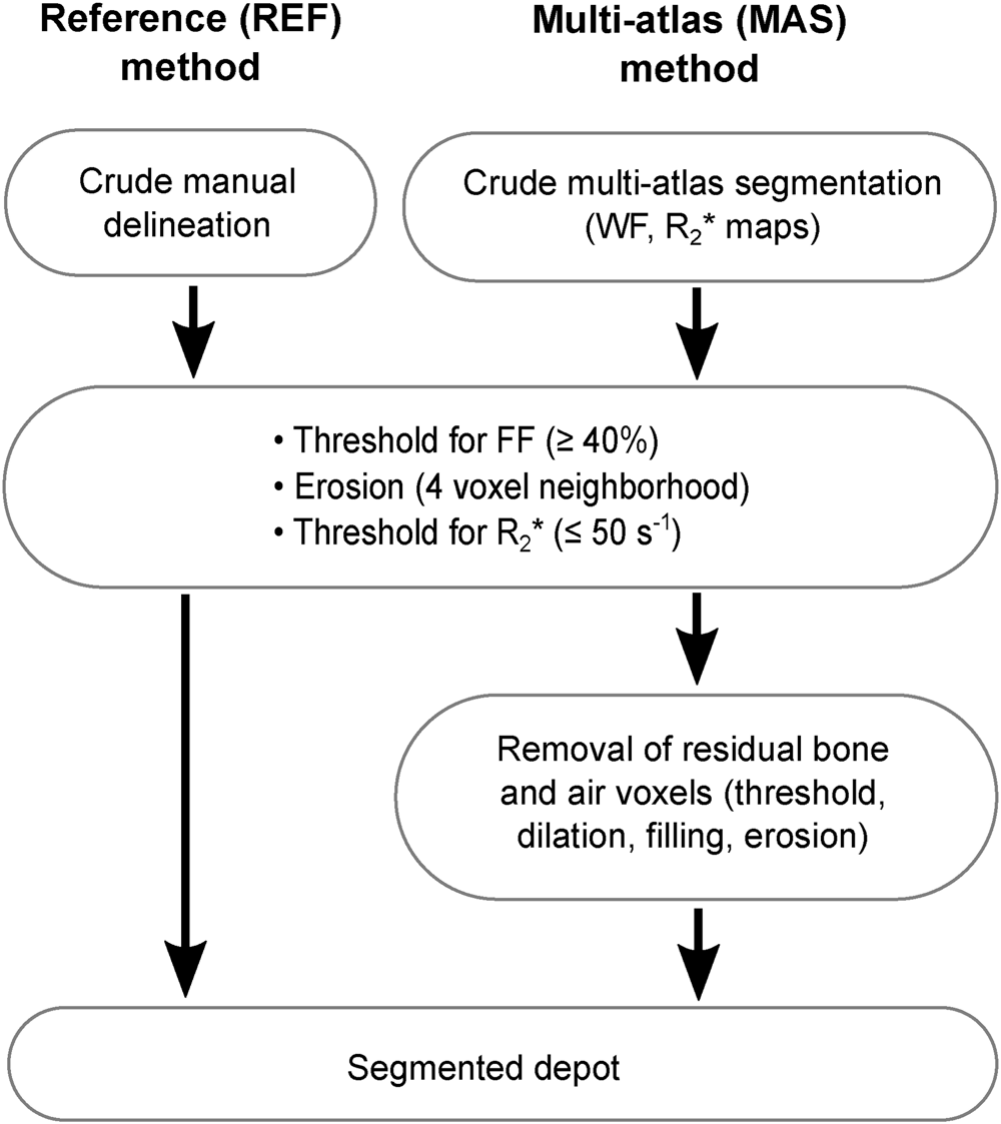
Flow charts of the semi-automated reference segmentation (REF) method and the automated multi-atlas segmentation (MAS) method. In the MAS method, the initial crude manual delineation of the REF method was replaced by a crude multi-atlas segmentation. Thereafter, a common set of automated fine adjustments was applied to the two methods (thresholding, erosion). In the MAS method, the segmented depot was obtained after a refinement step where residual voxels of air and bone were removed (by thresholding, dilation, filling of holes, erosion). In the REF method, the segmented depot was obtained directly after the automated fine adjustments as voxels in bone and air were assumed to be avoided in the manual delineation.

**Figure 2:**
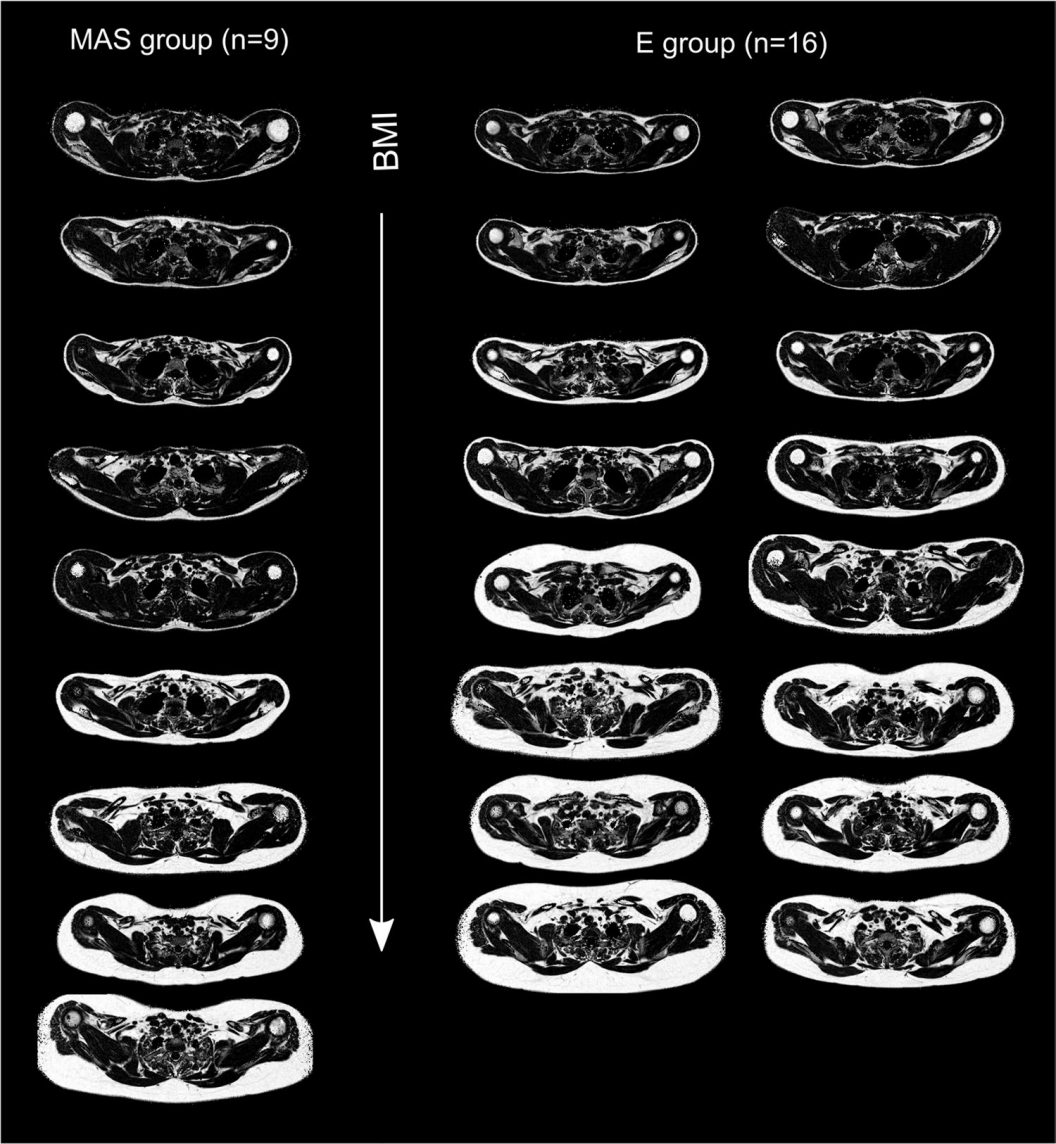
Axial fat fraction (FF) maps of the subjects in the multi-atlas segmentation (MAS) group and the evaluation (E) group. Both groups were composed of adults and children with body compositions ranging from lean to obese, here ordered according to BMI.

In the present study, an automated MAS method was developed and subsequently validated by a semi-automated reference (REF) method. An overview of the two methods is presented by the flow chart in Fig. 1 and methodological details are provided in the following subsections. The subjects used for producing and evaluating the MAS method were divided into two groups with similar characteristics: the MAS group and the evaluation (E) group (illustrated in Fig. 2). The MAS group formed an essential part of the MAS method and the E group was used for validating the MAS method with the REF method.

### Subjects

Seventeen children and adolescents from the *Beta*-*cell function in Juvenile Diabetes and Obesity* project and eight adults from previous MR method development studies, approved by the Regional Ethical Review Board in Uppsala, were included in this study. All participants had given informed written consent to participate and the procedures were carried out in accordance with the relevant guidelines and regulations. The subjects were divided into an MAS group of 9 subjects [five males, four females, age: 23 ± 10 years (mean ± standard deviation), age range: 12–37 years, body mass index (BMI): 26.1 ± 5.7 kg/m^2^] and an E group of 16 subjects [five males, eleven females, age: 17 ± 7 years, age range: 11–33 years, BMI: 27.7 ± 6.4 kg/m^2^]. Both groups were composed of adult and child subjects with a wide range of body composition (Fig. 2). As the performance of the MAS method was directly influenced by the MAS group, only subjects with relatively high image quality (subjective assessment mainly considering motion artefacts) were included in this group. The number of subjects in the MAS and E groups was considered appropriate in view of similar work published previously.

### Image acquisition and reconstruction

Water-fat MRI of the cervical-supraclavicular depot was performed on a clinical whole-body 1.5 T MR system (Philips Healthcare, Best, The Netherlands). Water images, fat images, FF maps and R_2_^*^ maps (inherently co-registered as acquired simultaneously) were obtained through a previously described in-house 3D reconstruction method^12^. Imaging and reconstruction parameters for the water-fat data are summarized in Table 1.

**Table 1:** Summary of imaging and reconstruction parameters for the water-fat data.

Imaging parameter	Value
Pulse sequence	3D multi gradient echo sequence
Coil	16 channel neurovascular receive coil
Scan time	4 min 40 s
Orientation	Axial
Repetition time	32.7 ms
Echo time 1/Echo time spacing	1.68/2.87 ms
Number of echoes	6 (unipolar)
Flip angle	6°
Water-fat shift	0.25 voxels
Fold-over direction	AP
SENSE acceleration	1.5 (AP direction)
Field of view (RL × AP × FH)	480 × 200 × 50 mm^3^
Acquired/Reconstructed voxel size	1.0 × 1.0 × 2.0/1.0 × 1.0 × 2.0 mm^3^
Number of slices	25
Number of signal averages	2
**Water-fat reconstruction parameter**	**Value**
Fat spectral model	Nine-peak
R_2_^*^ estimation strategy	Single R_2_^*^ estimation (decoupled determination)
Regularization parameter (μ)	10

Data were acquired under shallow breathing to reduce motion artefacts. The flip angle was chosen small to reduce T_1_-weighting. No contrast agents or pharmacological agents were administered. Data were reconstructed using a previously described method^12^. RL = right-left, AP = anterior-posterior, FH = feet-head direction. The regularization parameter, μ, reflects the influence of assumed magnetic field spatial smoothness, and hence the influence of noise, in fitting of the water-fat MRI signal model to the data^12^.

### The semi-automated reference segmentation method

The REF method was based on crude manual delineation of the sBAT depot and applied to the water-fat data of all MAS and E (group) subjects. In the MAS subjects, manual delineation was performed in all slices. In the E subjects, manual delineation was performed in even numbered slices only, to reduce the associated time-consuming work. The segmentation of the MAS subjects formed a part of the MAS method itself whereas the segmentation of the E subjects was used as reference for evaluating the performance of the MAS method.

The REF method consisted of a slightly modified version of a previously published semi-automated method^22^. According to the REF method, the segmentation of sBAT was accomplished in two steps: 1) Manual delineation of a crude sBAT volume of interest (VOI) performed by an experienced operator guided by radiologists. 2) Automated fine adjustment of the sBAT VOI for extraction of AT and for final labelling of voxels as sBAT or non-sBAT voxels.

#### REF method, Step 1: Manual delineation

**Figure 3:**
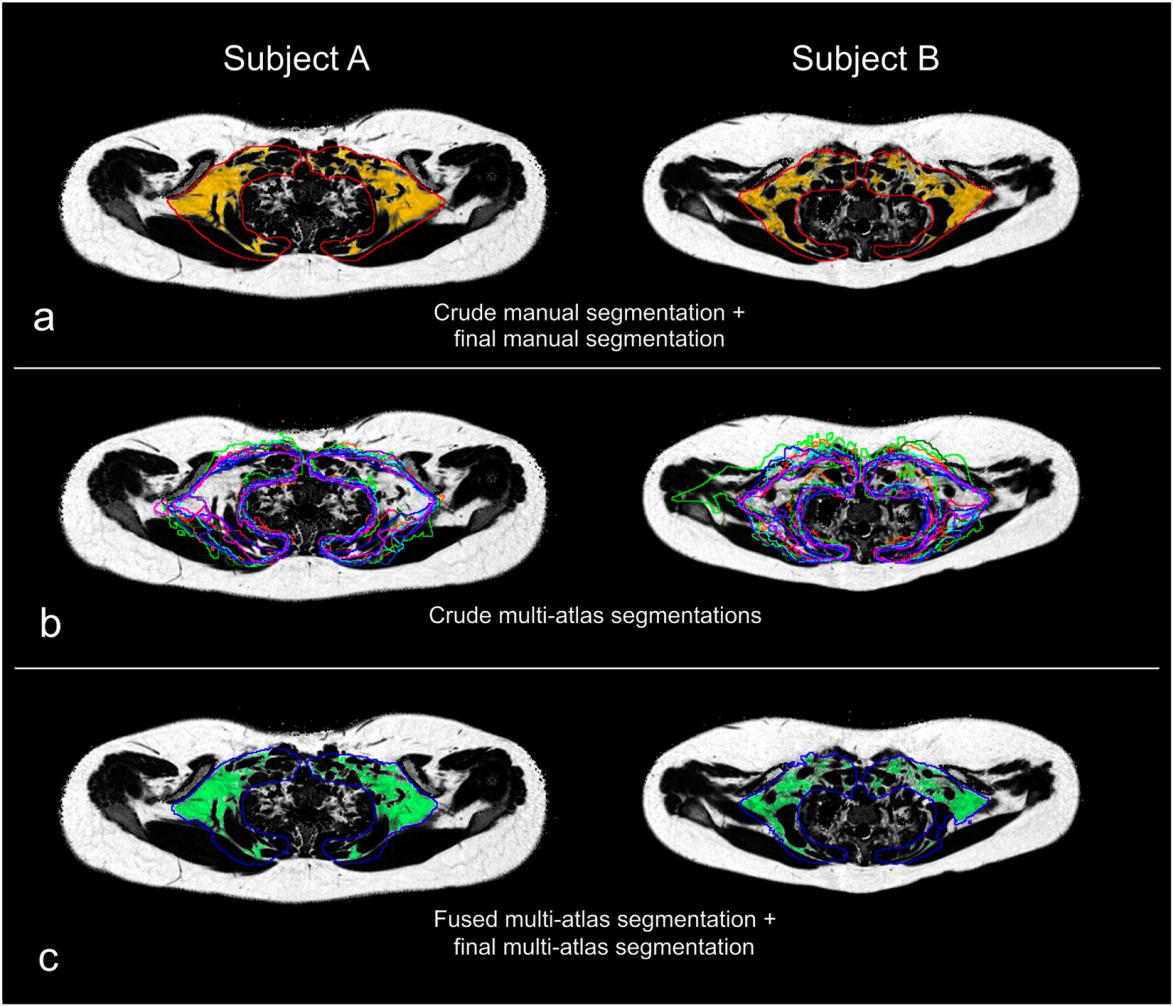
Examples of segmentation results from the semi-automated reference segmentation (REF) method and the automated multi-atlas segmentation (MAS) method. Axial fat fraction (FF) maps of two of the 16 evaluation (E) subjects A and B are overlaid with (**a**) the crude manual delineation (red contour) and the final manual segmentation after fine adjustment (sBAT_REF_, yellow area), (**b**) the crude multi-atlas segmentations (multi-colour contours) and (**c**) the fused crude multi-atlas segmentation (blue contour) and the final multi-atlas segmentation after fine adjustment and refinement (sBAT_MAS_, green area). Subject A: female, age = 16 years, BMI = 35.2 kg/m^2^. Subject B: female, age = 11 years, BMI = 28.6 kg/m^2^.

Segmentation of the crude sBAT VOI was performed manually and slice wise on the FF map meanwhile also considering the R_2_^*^ map and the water image for more accurate delineation of AT close to bone and lung. The crude sBAT VOI was defined, from anatomical criteria, to include AT located between the clavicula and the scapula and to exclude subcutaneous AT, bone marrow, and paravertebral fat (examples illustrated by the red contours in Fig. 3a). In regions where sBAT and subcutaneous AT were adjoining, the crude sBAT VOI was rather kept narrow than broad to reduce the infiltration of WAT. Additionally, care was taken to not include AT directly adjacent to the lungs and ribs as these areas were relatively difficult to delineate. In general, the exclusion of potential BAT voxels was considered as less severe than inclusion of distinct bone marrow or SAT voxels in the crude manual delineation.

#### REF method, Step 2: Automated fine adjustment of segmentation

Within the crude sBAT VOI, an automated segmentation (implemented in MATLAB) was applied to isolate the AT and concurrently decrease the partial volume effects from adjacent non-fatty tissues. The automated segmentation was accomplished by applying a voxel threshold on FF (≥40%) followed by subsequent 2D erosion with a four-neighbourhood structuring element (SE) and a voxel threshold on R_2_^*^ (≤50 s^−1^). The threshold limits were determined empirically and aimed at excluding non-fatty tissue and probable border voxels. The final segmented sBAT region (examples illustrated by the yellow areas in Fig. 3a) is hereafter denoted sBAT_REF_ and the associated mean FF and R_2_^*^ are denoted ${\overline{{\rm{FF}}}}_{{\rm{REF}}}\,{\rm{and}}\,{\overline{{{\rm{R}}}_{2}^{\ast }}}_{{\rm{REF}}}$, respectively.

### The automated multi-atlas segmentation method

MAS methods are based on the use of previously segmented images (atlases) for subsequent segmentation of novel, to-be-segmented images (targets)^19^. MAS methods are generally automated and based on pairwise image registration between each atlas and target, where the atlases and targets can be represented by e.g. 2D or 3D images. In this project, the atlases were generated from the MAS subject water-fat data to which crude manual segmentations were associated. The targets were represented by the corresponding unsegmented water-fat data of the E subjects.

MAS of a single target was accomplished by the following steps (implemented in MATLAB): (1) Generation of atlases by preprocessing the input images to the image registration and by manual delineation of the crude sBAT VOIs. (2) Image registration-driven deformation of each atlas for anatomical matching to the target and for determining the spatial correspondence (deformation field) between the atlas and the target. (3) Propagation of the crude atlas segmentations to the target by applying the deformation field. (4) Fusion of the propagated segmentations, one for each atlas, for obtaining a single merged crude segmentation of the target. (5) Fine adjustment and refinement of the fused crude segmentation leading to a final segmentation of the target.

#### MAS method, Step 1: Generation of atlases

Each atlas consisted of a water fraction (WF) and R_2_^*^ map with an associated crude sBAT VOI segmentation. The WF and R_2_^*^ maps were chosen as they were both quantitative (i.e. directly comparable between subjects) and reflected different body tissue properties. The WF map was preferred to the FF map as it could be preprocessed into an apparent lean tissue body. This was due to the low WF of AT that enabled the tissue to be interpreted as background by the computer algorithm. The lean tissue body nearly enclosed the sBAT depot and was of relatively high inter-subject anatomical similarity, despite a wide range of body fat content, and was therefore suitable for the image registration in the MAS method.

**Figure 4:**
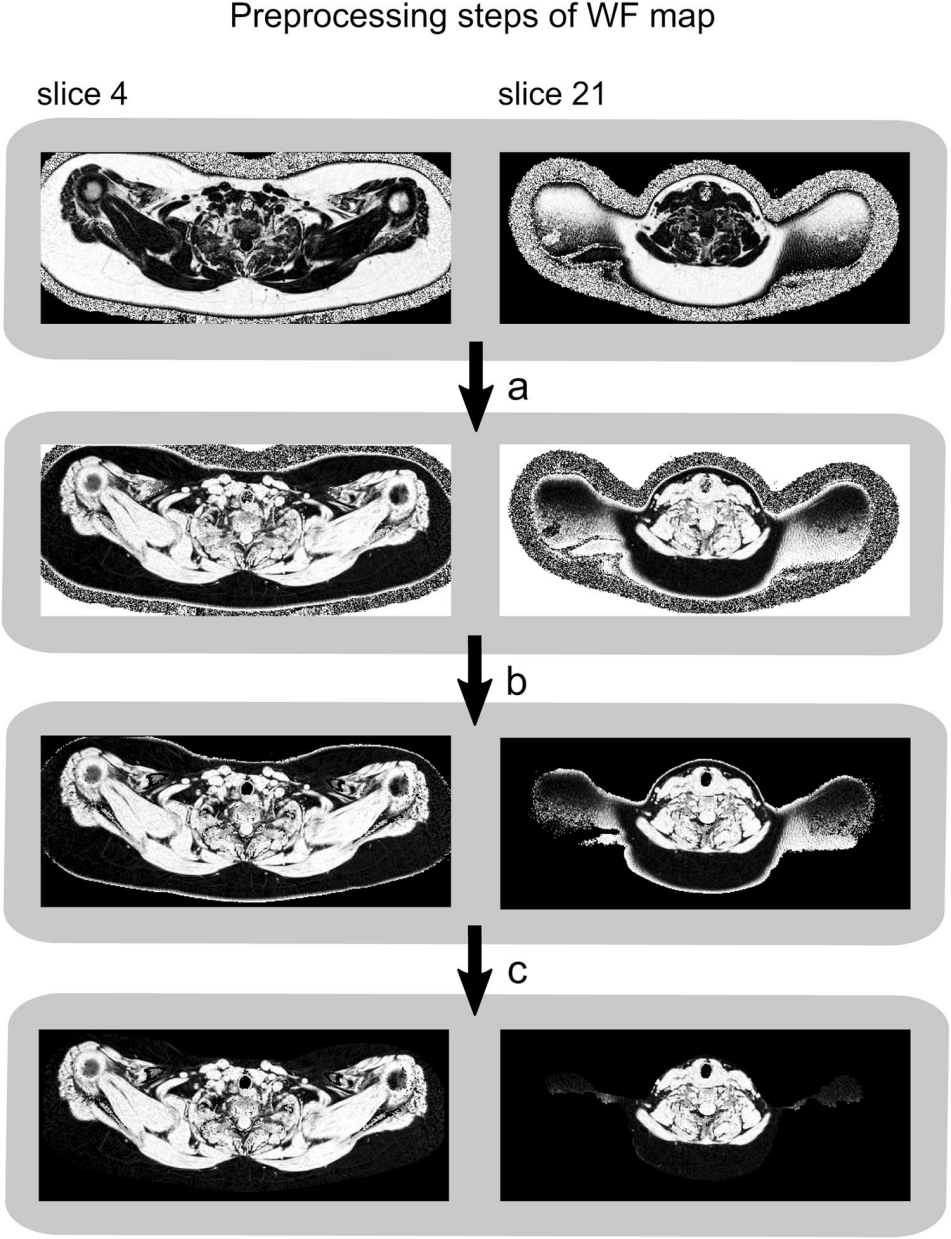
The preprocessing steps applied to the water fraction (WF) map prior to image registration in the multi-atlas segmentation (MAS) method. Two slices of the same subject are illustrated. Step a: Calculation of the WF map from the fat fraction (FF) map. Step b: Removal of background voxels. Step c: Removal of skin voxels. After preprocessing, the WF map consisted of an apparent lean tissue body. As the preprocessed WF map nearly surrounded the cervical-supraclavicular (sBAT) depot and exhibited relatively low inter-subject variability, it was suitable for registration purposes.

**Figure 5:**
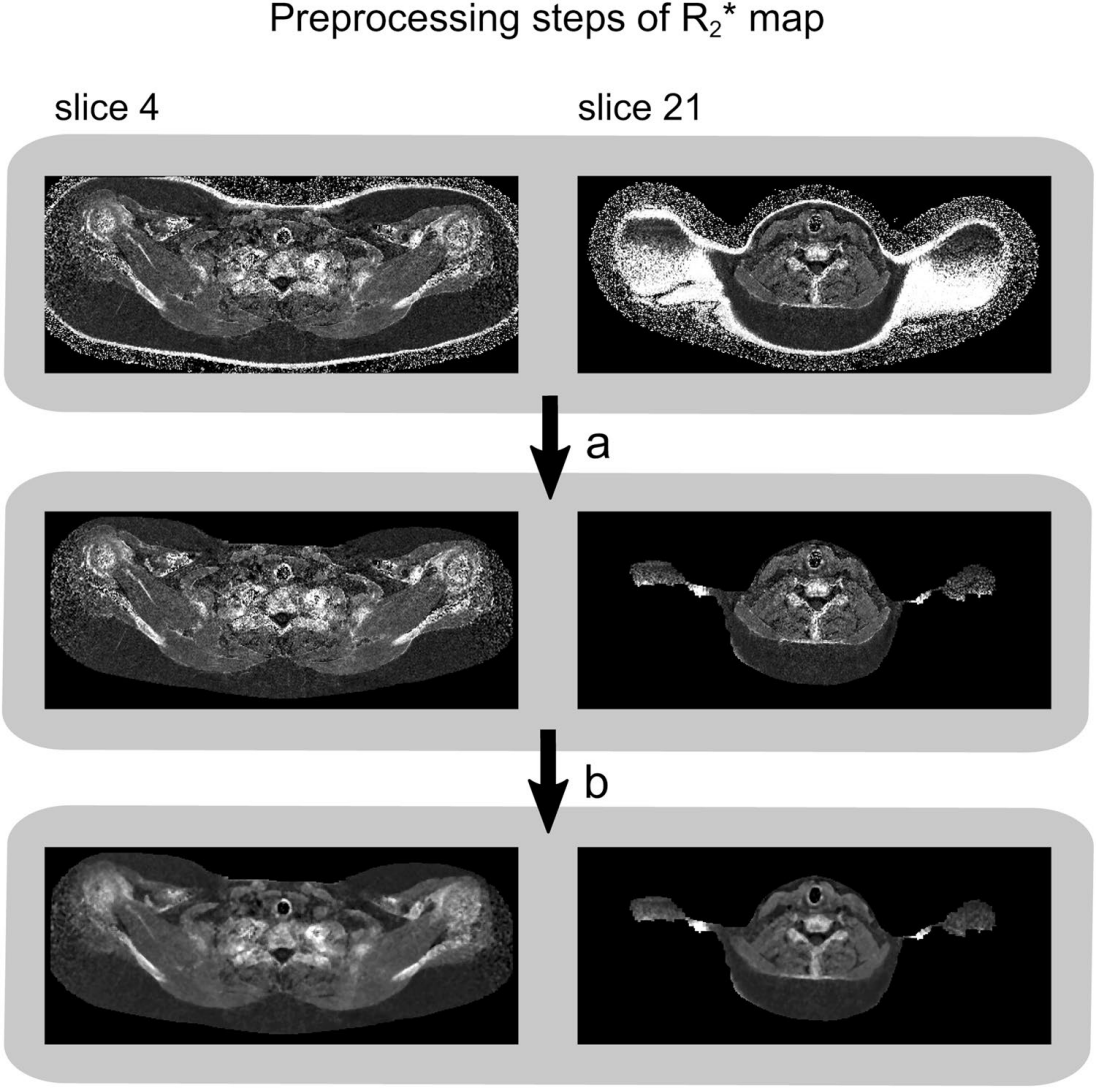
The preprocessing steps applied to the R_2_^*^ map prior to image registration in the multi-atlas segmentation (MAS) method. Two slices of the same subject are illustrated. Step a: Removal of background voxels outside of the body and skin voxels. Step b: Reduction of image noise using a median-filter. The preprocessed R_2_^*^ map exhibited high voxel intensities in areas close to bone and air-filled structures, which guided the matching of these anatomies under image registration.

The preprocessing steps of the WF map (illustrated in Fig. 4) were as follows: (a) Voxel wise computation of the WF map (WF = 1 − FF) (b) Removal of background (i.e. voxels containing air and cortical bone), represented by noisy areas in the WF map due to the low water and fat signal. The removal was accomplished by voxel wise thresholding on the sum of the water and fat signal. (c) Removal of skin voxels by exploiting its tissue specific FF and R_2_^*^ properties and anatomical location, followed by a series of erosion, dilation and filling steps. The major steps were as follows: A 2D erosion (four-neighbourhood SE), applied iteratively on axial slices under constraints on FF (≤30%) and R_2_^*^ (≥80 s^−1^) for eliminating one voxel-layer of skin at a time (thresholds empirically determined). In detail, the iterative process allowed removal of thin and thick layers of skin (i.e. voxels of low FF and high R_2_^*^) without substantially affecting adipose tissue (i.e. voxels of high FF and low R_2_^*^) or muscle tissue (i.e. voxels of low FF and R_2_^*^). For examples of tissue specific FF and R_2_^*^ properties, see Figs 4 and 5. Then, residual clusters of unwanted voxels in skin or outside of the body were omitted by considering only the largest connected component. Subsequent 2D morphological closing (5 × 5 SE), with intermediate filling of holes, was applied to correct rare cases of erosion underneath the skin. Finally, 2D erosion (7 × 7 SE) without constraints was applied to further decrease the amount of any residual skin voxels.

The R_2_^*^ map was preprocessed into an image exhibiting high voxel intensities in areas close to bone and air-filled structures and used to guide the matching of these anatomies under image registration. The preprocessing steps (illustrated in Fig. 5) were as follows: (a) Removal of skin (and simultaneously also background outside of the body) by applying the same methods as described for the WF map. (b) Smoothing of the R_2_^*^ map by applying a median-filter (3 × 3 × 3 SE) for noise-reduction.

The atlas segmentation was conducted by manual delineation, largely in accordance with the first step of the REF method. But in addition to appropriate delineation of AT, care was taken to also include lean tissue in areas that might correspond to sBAT in other subjects with a different body composition. When images of different subjects were registered, the lean tissue bodies and bone structures were expected to be adequately aligned whereas locally within the sBAT region (i.e. between the lungs, clavicula and scapula), suboptimal matching between lean tissue areas and AT areas was common. In the sBAT region of a lean subject, the amount of lean tissue content was typically higher than the amount of AT. In an obese subject, the relative amounts were generally the opposite. As a consequence, a lean MAS subject was expected to underestimate the sBAT depot coverage of an obese E subject unless the crude sBAT VOI of the MAS subject also included lean tissue within the sBAT region.

#### MAS method, Step 2: Image registration

Prior to image registration, the WF and R_2_^*^ maps of the E subjects were preprocessed according to the same procedure as described above (section *Generation of atlases*). Pairwise image registration between each atlas and each target was accomplished in two steps by the ITK-based Elastix software^23^. In the first step, the atlas WF map (moving image) underwent affine deformation to match the target WF map (fixed image) during optimization of the normalized correlation (optimizer = adaptive stochastic gradient descent, number of resolutions = 4, max number of iterations = 3000, b-spline interpolation order = 1). The affinely registered dataset was then used as starting point for the second step where a weighted sum (weight = 1:1) of the normalized correlation between the atlas and target WF and R_2_^*^ maps was optimized under an elastic deformation (b-spline transform, optimizer = adaptive stochastic gradient descent, number of resolutions = 4, max number of iterations = 7000, b-spline interpolation order = 1, final grid spacing = 20 mm). Parameter settings were chosen to provide an appropriate trade-off between computational cost and degree of elasticity in the allowed deformations.

#### MAS method, Step 3: Propagation of segmentations

For each pairwise image registration, the resulting deformation field was applied to transfer the crude sBAT VOI from the atlas to the target. Linear interpolation, preserving the voxel coverage values, was used as propagation strategy.

#### MAS method, Step 4: Fusion of segmentations

For each target, nine different crude segmentations were obtained (i.e. one from each atlas, examples illustrated by the multi-colour contours in Fig. 3b). These segmentations were combined, by majority voting, to a single and combined crude segmentation of the target (examples illustrated by the blue contours in Fig. 3c). For a voxel to be labelled as a crude sBAT VOI voxel, it had to be labelled as such by more than half of the atlases. As the imaged volume only partially covered the cervical-supraclavicular depot in the superior-inferior direction and as the shoulder area is flexible and sensitive to subject positioning, the imaged volume was slightly differently located (with respect to anatomy) in different subjects. Due to this inter-subject shift of the imaged volume, the majority voting of a specific voxel was performed only among the atlases in which the voxel was represented. With this approach, the influence from subject positioning on the voxel label was reduced. In addition, this modified majority voting prevented unnecessary exclusion of sBAT voxels due to shift in depot coverage between the subjects.

#### MAS method, Step 4: Fine adjustment and refinement of segmentation

Extraction of AT within the crude sBAT VOI was performed automatically according to the second step of the REF method. In a final refinement step, an attempt of removing any residual voxels within air and bone was made by applying a threshold on the total water and fat signal with additional dilation, filling of holes and erosion. The final segmented sBAT region (examples illustrated by the green areas in Fig. 3c) is here denoted sBAT_MAS_ and the associated mean FF and R_2_^*^ are denoted ${\overline{{\rm{FF}}}}_{{\rm{MAS}}}\,{\rm{and}}\,{\overline{{{\rm{R}}}_{2}^{\ast }}}_{{\rm{MAS}}}$, respectively.

### Evaluation of the MAS and REF methods

The MAS and REF methods were compared with respect to segmentation overlap and correspondence in estimated $\overline{{\rm{FF}}},\overline{{{\rm{R}}}_{2}^{\ast }}$ and sBAT volume. Evaluation was performed in even numbered slices only as the segmentation of the E subjects, according to the REF method, was limited to these slices. The MAS method computation time was estimated per target.

The Dice similarity coefficient (Dice) was used to calculate the overlap between the sBAT segmentations according to

1 $${\rm{D}}{\rm{i}}{\rm{c}}{\rm{e}}=2\cdot \frac{|{{\rm{s}}{\rm{B}}{\rm{A}}{\rm{T}}}_{{\rm{M}}{\rm{A}}{\rm{S}}}\cap {{\rm{s}}{\rm{B}}{\rm{A}}{\rm{T}}}_{{\rm{R}}{\rm{E}}{\rm{F}}}|}{|{{\rm{s}}{\rm{B}}{\rm{A}}{\rm{T}}}_{{\rm{M}}{\rm{A}}{\rm{S}}}|+|{{\rm{s}}{\rm{B}}{\rm{A}}{\rm{T}}}_{{\rm{R}}{\rm{E}}{\rm{F}}}|}=\frac{2{{\rm{n}}}_{{\rm{T}}{\rm{P}}}}{2{{\rm{n}}}_{{\rm{T}}{\rm{P}}}+{{\rm{n}}}_{{\rm{F}}{\rm{P}}}+{{\rm{n}}}_{{\rm{F}}{\rm{N}}}},$$

where n_TP_, n_FP_ and n_FN_ represent the number of true positive (TP), false positive (FP) and false negative (FN) voxels, respectively. Voxels within the imaged volume of the E subject but outside the imaged volume of all MAS subjects were discarded in the Dice calculation (i.e. not considered as FN voxels). This was due to the interpretation of these non-overlapping voxels to be results of suboptimal subject positioning rather than of suboptimal MAS method performance. Based on the same argument, the segmented sBAT_REF_ volume was defined as

2 $${{\rm{V}}}_{{\rm{REF}}}=({{\rm{n}}}_{{\rm{TP}}}+{{\rm{n}}}_{{\rm{FN}}})\cdot {\rm{voxel}}\,{\rm{volume}},$$

where non-overlapping voxels were omitted. The segmented sBAT_MAS_ volume was defined as

3 $${{\rm{V}}}_{{\rm{MAS}}}=({{\rm{n}}}_{{\rm{TP}}}+{{\rm{n}}}_{{\rm{FP}}})\cdot {\rm{voxel}}\,{\rm{volume}},$$

The FP and FN voxels were evaluated for systematic patterns. The FP voxels were visually categorized as distinct SAT, distinct bone marrow or non-specific AT voxels. The non-specific AT voxels included those adjacent to SAT (but which could not unambiguously be determined as SAT), to lungs and bone and to other borders of sBAT_REF_.

The difference in $\overline{{\rm{FF}}}$ estimations, between the MAS and REF methods, was calculated as ${\rm{\Delta }}\overline{{\rm{FF}}}={\overline{{\rm{FF}}}}_{{\rm{MAS}}}-{\overline{{\rm{FF}}}}_{{\rm{REF}}}$. The difference in other quantitative parameters were calculated and denoted accordingly. Differences in FF were expressed in percentage points (pp). Linear regression was used to evaluate the association between the quantitative parameters obtained by the two methods. Linear regression was also used to evaluate the association of any MAS method bias with measurement results and subject characteristics. Mean values were compared by Student’s paired t-test using a two-tailed distribution. The approximate normal distribution of the data was assessed by visual inspection.

The repeatability of the REF method was evaluated by letting the same operator repeat the manual delineation of the crude sBAT VOI, in even numbered slices of six of the E subjects, approximately two months after the original delineation. The repeatability was determined by means of the Dice (equation 1).

Statistical analysis was performed using Statistica 12 and 13 (StatSoft Scandinavia AB, Uppsala, Sweden) for Windows and Excel for Mac 2011. Reported data were expressed as means ± standard deviations (SD) and ranges. P values < 0.05 were considered statistically significant. The significance level was not adjusted for multiple comparisons.

### Code and data availability

Currently the computer code is not available online but could be obtained upon request. MRI data in image format is not available online as this has not explicitly been authorized by the Regional Ethical Review Board.

## Results

**Figure 6:**
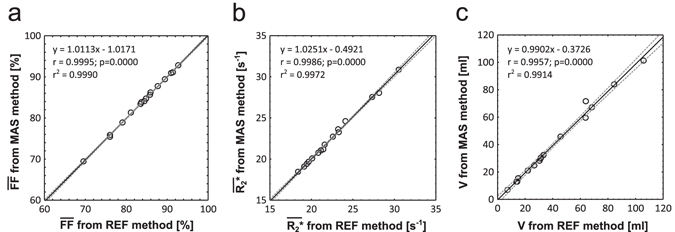
Correspondence in fat fraction, R_2_^*^ and volume measurements. Significant correlations observed between (**a**) mean fat fraction ($\overline{{\rm{FF}}}$), (**b**) mean R_2_^*^ ($\overline{{{\rm{R}}}_{2}^{\ast }}$) and (**c**) segmented volume (V), of the cervical-supraclavicular depot (sBAT) as obtained by the multi-atlas segmentation (MAS) method and the semi-automated reference segmentation (REF) method. Dashed lines indicate the 95% confidence interval for the regression line. Number of data points (subjects) = 16. The significance level was not adjusted for multiple comparisons.

The results from segmentation of sBAT, according to the MAS and REF methods, are summarized in Table 2. The correspondence between the two methods, with respect to estimations of $\overline{{\rm{FF}}},\overline{{{\rm{R}}}_{2}^{\ast }}$ and segmented volumes, is graphically presented in Fig. 6. Examples of segmentation performance of the two methods are visually presented in single slices of two E subjects in Fig. 3. The computation time for the MAS method was estimated to approximately 0.5 hours per E subject (3.40 GHz processor, 16.0 GB RAM, without parallelization). When considering manual crude delineation in all 25 slices, the duration of the REF method was approximately 1 hour of which the automated fine adjustments accounted for a negligible part (of the order seconds).

**Table 2:** Summary of segmentation results.

Segmentation method	$\overline{{\bf{FF}}}$ [%]	$\overline{{{\bf{R}}}_{{\bf{2}}}^{{\boldsymbol{\ast }}}}\,[{{\bf{s}}}^{-{\bf{1}}}]$	V [ml]	Dice [%]
REF	83.9 ± 6.3	22.6 ± 3.5	41.1 ± 28.4	92.5 ± 2.7 (88.1–96.4)
	(69.6–92.7)	(18.4–30.5)	(7.2–105.8)	
MAS	83.8 ± 6.4	22.6 ± 3.6	40.3 ± 28.3	
	(69.5–92.8)	(18.5–30.9)	(7.0–101.3)	

Data on cervical-supraclavicular adipose tissue (considered as suspected brown adipose tissue, denoted sBAT). Measurements of mean fat fraction $(\overline{{\rm{FF}}})$, mean R_2_^*^ $(\overline{{{\rm{R}}}_{2}^{\ast }})$ and volume (V) of segmented sBAT, obtained by the multi-atlas segmentation (MAS) method and the semi-automated reference segmentation (REF) method. Segmentation overlap was estimated by the dice similarity coefficient (Dice). Measurements reported as group means ± standard deviations (ranges) of individual means.

### Segmentation overlap

The segmentation overlap between the MAS and REF methods, as evaluated within the E group, was obtained to Dice = 0.93 ± 0.03 (0.88–0.96, Table 2). The performance of the two methods is illustrated in single slices of the E subjects A and B in Fig. 3, where subject A obtained the highest Dice (Dice = 0.96) and subject B the lowest (Dice = 0.88). Most FP voxels were located in non-specific AT, especially along the diffuse border between distinct SAT and sBAT_REF_. A smaller portion of the FP voxels were located in distinct SAT and bone marrow. The FN voxels were generally scattered around the sBAT_REF_ border without any obvious direction of preference.

### Mean FF, R_2_* and volume estimations

The $\overline{{\rm{FF}}}$s, obtained by the MAS and REF methods, were observed to correlate (r > 0.99, P < 0.0001, Fig. 6a). Correlations were also observed between the corresponding $\overline{{{\rm{R}}}_{2}^{\ast }}$s (r > 0.99, P < 0.0001, Fig. 6b) and segmented volumes (r > 0.99, P < 0.0001, Fig. 6c). There was no difference observed in $\overline{{\rm{FF}}}$ (−0.07 ± 0.21 pp, P = 0.23), $\overline{{{\rm{R}}}_{2}^{\ast }}$ (0.07 ± 0.21 s^−1^, P = 0.17) or segmented volume (−0.77 ± 2.64 ml, P = 0.26) between the two methods.

### Association of bias with measurement results and subject characteristics

A trend of a positive correlation between ${\rm{\Delta }}\overline{{{\rm{R}}}_{2}^{\ast }}$ and ${\overline{{{\rm{R}}}_{2}^{\ast }}}_{{\rm{REF}}}$ (r = 0.42, P = 0.10) was observed but no associations between either ${\rm{\Delta }}\overline{{\rm{FF}}}$ and ${\overline{{\rm{FF}}}}_{{\rm{REF}}}$ (r = 0.34, P = 0.20) or ΔV and V_REF_ (r = −0.11, P = 0.70) were obtained. Dice was observed to be positively correlated with BMI (r = 0.54, P = 0.03) and there was a trend of positive correlation between Dice and V_REF_ (r = 0.47, P = 0.07). However, there was no observed association between Dice and age (r = 0.09, P = 0.73). No association was observed between ${\rm{\Delta }}\overline{{\rm{FF}}}$ and either one of BMI (r = 0.23, P = 0.40), V_REF_ (r = 0.20, P = 0.45) and age (r = 0.19, P = 0.49). There were trends of negative correlation between ${\rm{\Delta }}\overline{{{\rm{R}}}_{2}^{\ast }}$ and each one of BMI (r = −0.43, P = 0.09) and V_REF_ (r = −0.38, P = 0.15) but no association between ${\rm{\Delta }}\overline{{{\rm{R}}}_{2}^{\ast }}$ and age (r = −0.16, P = 0.55).

### Repeatability of the reference method

The overlap between two repeated segmentations by the REF method, as evaluated within a subcohort of the E group, was larger [Dice = 0.94 ± 0.02 (0.91–0.97)] than that obtained from the MAS and REF method comparison [Dice = 0.91 ± 0.02 (0.89–0.95)] (P < 0.01).

## Discussion

In the present work, a multi-atlas based method for segmentation of human sBAT from water-fat MR images has been described and evaluated. This MAS method is fully automated, time-efficient and provides objective results similar to those obtained by a semi-automated REF method based on manual delineation. When compared to the REF method, the MAS method demonstrated a relatively large segmentation overlap (Dice = 0.93 ± 0.03) with sufficient robustness (Dice range: 0.88–0.96) in lean to obese adults and children. Discrepancies in segmentations obtained with the MAS and REF methods corresponded to inclusion of only small amounts of undesirable SAT and bone marrow. Moreover, the discrepancy in segmentation overlap did not result in significant differences in $\overline{{\rm{FF}}},\overline{{{\rm{R}}}_{2}^{\ast }}$ or volume estimations. The relatively large standard deviations in the segmented volumes (Table 2) reflected the large variation in body composition between the E subjects.

The excellent correlations (r ≈ 0.99) and agreements (slope k ≈ 1) between the MAS and REF methods, with respect to estimations of $\overline{{\rm{FF}}},\overline{{{\rm{R}}}_{2}^{\ast }}$ and segmented volumes, suggest the latter method to be replaceable by the former without loss of accuracy. When investigating the association of MAS method bias with measurement results, the trend of positive correlation between ${\rm{\Delta }}\overline{{{\rm{R}}}_{2}^{\ast }}$ and ${\overline{{{\rm{R}}}_{2}^{\ast }}}_{{\rm{REF}}}$ indicated an $\overline{{{\rm{R}}}_{2}^{\ast }}$-dependence leading to accurate estimation of $\overline{{{\rm{R}}}_{2}^{\ast }}$ ≈ 20 s^−1^ but 1% overestimation of $\overline{{{\rm{R}}}_{2}^{\ast }}$ ≈ 30 s^−1^ by the MAS method. Moreover, the positive correlation between Dice and BMI and the trend of positive correlation between Dice and segmented volume (V_REF_) suggest inferior segmentation overlap in lean as compared to obese subjects. This is probably due to a higher segmented surface-to-volume ratio in lean subjects, which is associated with a smaller segmentation overlap. The stronger association of Dice with BMI than with segmented volume (V_REF_) was somewhat surprising as volume is expected to more directly reflect the surface-to-volume ratio. Separate and dedicated sets of lean-specific and obese-specific atlases could likely improve the segmentation of lean and obese subjects, respectively. However, a common set of lean and obese atlases is preferable for objective and accurate measurements across a wide range of body composition, also including e.g. slightly overweight subjects. An alternative future approach to using separate sets of atlases is to refine the segmentation fusion method. Instead of majority voting, where each atlas segmentation is weighted equally, a weighted voting approach might improve the results by smoothly adjusting the influence of individual atlases with respect to their similarities to the target^19^. Despite the inclusion of both children and adults (11–33 years), there was no observed association between Dice and age. Although the segmentation overlap was found to vary with subject body composition it did not introduce any BMI- or volume-associated bias in $\overline{{\rm{FF}}}$. However, a trend of negatively correlated ${\rm{\Delta }}\overline{{{\rm{R}}}_{2}^{\ast }}$ with BMI and segmented volume (V_REF_) was observed and might lead to biased results, e.g. in cross-sectional studies including both lean and obese subjects. To exemplify the effect of this trend, the $\overline{{{\rm{R}}}_{2}^{\ast }}$ of a subject with BMI ≈ 20 kg/m^2^ would be overestimated by approximately 0.2 s^−1^ by the MAS method whereas the $\overline{{{\rm{R}}}_{2}^{\ast }}$ of a subject with BMI ≈ 33 kg/m^2^ would be estimated accurately. The potential over- or underestimation related to the segmented volumes would be approximately up to ± 0.2 s^–^^1^. Age-associated bias in $\overline{{\rm{FF}}}$ and $\overline{{{\rm{R}}}_{2}^{\ast }}$ was not observed and may be considered as negligible. In conclusion, possible errors related to $\overline{{{\rm{R}}}_{2}^{\ast }}$ measurements and body composition are likely to be small compared to other sources of errors.

Most of the FP voxels were observed to be located in non-specific AT and only smaller amounts in distinct SAT and bone marrow. As the border between sBAT and SAT is not well-defined, AT near the border is more likely than distinct SAT to contain considerable clusters of BAT. Consequently, the mislabelling of non-distinct AT was considered as less severe than that of distinct SAT and bone marrow.

The segmentation overlap was observed to be larger between two repeated executions of the REF method than between the MAS and REF methods. This result was probably influenced by the large heterogeneity in subject body composition, which limited the performance of the MAS method. Nevertheless, the results were comparable and this drawback with the MAS method should be considered in light of the time efficiency advantage.

It should be noted that neither the REF nor the MAS segmentations of sBAT refer to AT verified to be BAT on a subject specific basis. However, the probability of the segmentations to be or to contain BAT is relatively high in the age span of subjects in the present study^15, 24^. The not well-defined shape of the sBAT depot makes the manual delineation, used for creating atlases and reference segmentations, challenging and operator-dependent. This difficulty was reflected in the REF method repeatability (Dice = 0.94), which nevertheless was relatively high. However, an advantage with choosing a multi-atlas approach for the automated method is that the studied volume easily can be adjusted by changing the manual delineation of the atlases, without editing the algorithm. Besides ambiguous borders, the mixed content of BAT and WAT in the sBAT depot^18, 25^ results in partial volume effects that prevent true BAT segmentation with the limited resolution of current imaging modalities. In contrast, the anatomically well-defined and relatively pure rodent iBAT depot is more easily identified, less affected by partial volume effects and less challenging to segment. Automated methods for segmenting rodent iBAT in MR images have been presented^26, 27^. Although adaption for future use in humans was suggested by the authors, the adjustments are not trivial due to the aforementioned differences between the rodent iBAT and the human sBAT depots. To our knowledge, no automated method for inter-subject segmentation of human BAT depots in MRI-only data has been presented. In a bi-modal study, based on both FDG-PET/CT and water-fat MRI^14^, segmentation of active BAT was accomplished by a similar method to those applied for standalone PET/CT studies. Subsequently, intra-subject registration of MR and PET/CT images was used to transfer BAT segmentations to the MRI data, thereby avoiding manual delineation of the MR images. Bi-modal studies could potentially be used for establishing MRI properties of BAT, anatomically defined from PET/CT guidance, with the future goal to estimate BAT amount and activity solely with MRI. However, further development is required. With the increasing availability of integrated PET/MR systems, the conditions for PET-guided automated segmentation of BAT will likely be improved due to simultaneous acquisition of the PET and MR data.

Apart from difficulties in separating sBAT from WAT, the imaged shoulder region is flexible and non-rigid and thereby challenging for the registration step of the MAS method. However, the two-step image registration procedure (affine deformation prior to elastic deformation) was shown to sufficiently align the anatomies. Moreover, the inter-subject heterogeneity in body composition and size (due to inclusion of both adult and child subjects) further increased the complexity of the registration task. To improve the segmentation results, a group of subjects representative of the whole cohort were chosen for the MAS group. To further improve the registration outcome, some preprocessing steps were applied to the input images, e.g. removal of skin voxels. As the skin voxels were located at distances from the lean tissue bodies that differed between subjects, depending on the amount of SAT, the removal was performed to avoid unpredictable image registration distortions. Another measure that probably improved the image registration performance was the standardized subject positioning which likely led to smaller deformations and more robust results.

Another challenge in the present study was that of bone detection during manual delineation. The appearance of bone in water-fat images of children differs from that of adults due to disparities in cortical bone thickness and bone marrow content. As a consequence, the ability to detect bone was generally perceived as more difficult in children.

Due to imaging time constraints, there was only partial sBAT depot coverage (5 cm in the superior-inferior direction). Therefore, the positioning of the image volume had a large impact on the sBAT overlap between different subjects. In order to use as much of the imaged sBAT depot as possible, the fusion of the individual atlas segmentations was based on a modified version of majority voting. According to this version, the voting of a specific voxel was performed only among the atlases for which the image volume overlapped with the voxel. A drawback with this approach was that the voxel labelling was performed by a number of atlases that varied from voxel to voxel, without considering the registration performance of the atlases. There could have been cases where the voting was performed only among a few atlases with suboptimal registrations, which could have resulted in suboptimal segmentations. However, this limitation was regarded as being related to imaging procedure and therefore not considered in the evaluation of the MAS method. A related limitation was that Dice and V_REF_ were calculated only from overlapping image volumes, between the atlases and the target. As a consequence, a considerable portion of the target sBAT could have been omitted in the Dice calculation and the target V_REF_ was probably underestimated. Nevertheless, ${\overline{{\rm{FF}}}}_{{\rm{REF}}}$ and ${\overline{{{\rm{R}}}_{2}^{\ast }}}_{{\rm{REF}}}$ were estimated from the whole sBAT volume of the target (in even numbered slices). The automated segmentation method has been evaluated for data acquired using a specific set of imaging parameters. The reliability of the method when applied to other data, e.g. those acquired with a different spatial coverage or resolution, has not been investigated. Although the method potentially could be satisfactory, such new data likely would require some adaption of the atlas data and/or computer algorithm for an optimal segmentation performance.

The performance of the automated segmentation method developed was similar to that of the semi-automated reference method. This result suggests the automated method to be suitable for time-efficient and objective measurements in large cohort research studies of brown adipose tissue (BAT).
